# Non-Typhoidal *Salmonella enterica* Bacteremia Complicated by Native Shoulder Septic Arthritis in a Patient with Sickle Cell Disease Following Foodborne Exposure: A Case Report and Literature Review

**DOI:** 10.3390/idr18020030

**Published:** 2026-04-02

**Authors:** Gabriel A. Godart, Vidit Yadav, Joseph M. Bestic, Bradley S. Schoch, Bryan D. Springer, Ravi V. Durvasula, Sammer M. Elwasila, Justin M. Oring

**Affiliations:** 1 Division of Infectious Diseases, Department of Medicine, Mayo Clinic, Jacksonville, FL 32224, USA; yadav.vidit@mayo.edu (V.Y.); durvasula.ravi@mayo.edu (R.V.D.); elwasila.sammer@mayo.edu (S.M.E.); oring.justin@mayo.edu (J.M.O.); 2 Department of Radiology, Mayo Clinic, Jacksonville, FL 32224, USA; bestic.joseph@mayo.edu; 3 Department of Orthopedic Surgery, Mayo Clinic, Jacksonville, FL 32224, USA; schoch.bradley@mayo.edu (B.S.S.); springer.bryan@mayo.edu (B.D.S.)

**Keywords:** non-typhoidal *Salmonella*, septic arthritis, sickle cell disease, *Salmonella enterica*, bacteremia, native shoulder joint infection

## Abstract

Background/Objectives: Non-typhoidal *Salmonella* (NTS) species are well-recognized causes of invasive infection in patients with sickle cell disease (SCD), with a particular predilection for the musculoskeletal system. Although *Salmonella* osteomyelitis is well described in this population, septic arthritis is uncommon, especially involving the shoulder joint. We describe a case of NTS bacteremia complicated by native shoulder septic arthritis in a patient with SCD and review its clinical implications. Methods: We report the clinical course, diagnostic evaluation, microbiologic findings, imaging studies, and management of a 22-year-old man with homozygous SCD who presented with a vaso-occlusive pain crisis and subsequently developed severe sepsis with persistent *Salmonella enterica* bacteremia following ingestion of undercooked poultry. Persistent bacteremia prompted further evaluation for metastatic infection using advanced imaging and diagnostic arthrocentesis. Results: Whole-body imaging identified septic arthritis of the native right shoulder, which was confirmed by synovial fluid cultures growing *Salmonella* species. The patient underwent arthroscopic irrigation and debridement for source control. Antimicrobial therapy was narrowed to intravenous ceftriaxone based on susceptibility data and continued for six weeks. The patient demonstrated clinical improvement with resolution of bacteremia and was discharged to rehabilitation to complete therapy. Conclusions: This case highlights the importance of a careful exposure history, including foodborne sources, in patients with SCD presenting with invasive *Salmonella* infection. Persistent bacteremia should prompt early investigation for metastatic foci, and timely surgical source control combined with targeted antimicrobial therapy is essential for optimal outcomes in this population.

## 1. Introduction

Sickle cell disease (SCD) is associated with a markedly increased susceptibility to invasive bacterial infections due to a combination of functional asplenia, impaired opsonization, complement dysfunction, and microvascular ischemic injury to bone and soft tissues [1,2,3]. Among bacterial pathogens, non-typhoidal *Salmonella* (NTS) species have long been recognized as important causes of invasive infection in patients with SCD, particularly involving the musculoskeletal system [2,4,5]. NTS osteomyelitis is well documented in this population and may account for a substantial proportion of osteoarticular infections in both pediatric and adult patients with SCD [4,5,6].

In contrast, septic arthritis caused by *Salmonella* species is considerably less common. Most reported cases involve weight-bearing joints such as the hip or knee and frequently occur in the presence of additional risk factors, including immunosuppression, systemic inflammatory disease, or prosthetic joints [7,8,9]. Although SCD is a well-established predisposing condition for *Salmonella* osteomyelitis, only a limited number of reports describe *Salmonella* septic arthritis in this population, and involvement of the native shoulder joint is particularly rare.

The pathogenesis of invasive *Salmonella* infection in SCD reflects a complex interaction between host immune dysfunction and bacterial virulence. Recurrent vaso-occlusion leads to intestinal mucosal injury and increased permeability, facilitating bacterial translocation from the gastrointestinal tract into the bloodstream [10,11,12]. Non-typhoidal *Salmonella* infections are most commonly acquired through foodborne exposure, particularly from undercooked poultry products, which represent a well-established reservoir for *Salmonella enterica* transmission [13]. Once bacteremia occurs, impaired reticuloendothelial clearance and functional asplenia allow sustained systemic dissemination and increase the risk of hematogenous seeding of susceptible tissues, including infarcted bone and adjacent joints [3,14].

Here, we report a case of persistent *Salmonella enterica* bacteremia complicated by hematogenous seeding of a native shoulder joint resulting in septic arthritis in a young adult with SCD. We also review the existing literature describing NTS septic arthritis and discuss the pathophysiologic mechanisms that predispose patients with SCD to invasive musculoskeletal infection.

## 2. Case Presentation

A 22-year-old man with homozygous SCD, complicated by functional asplenia and avascular necrosis of the right femoral head and bilateral humeral heads, presented in November with a vaso-occlusive pain crisis. He reported severe pain predominantly involving the right upper extremity and bilateral knees that was refractory to his home analgesic regimen. At presentation, he denied fever, gastrointestinal symptoms, respiratory complaints, or other focal signs of infection. He did report regular consumption of undercooked poultry before admission.

On hospital day 2, the patient developed acute clinical deterioration characterized by fever, rigors, sinus tachycardia, hypotension refractory to fluid bolus, and a new oxygen requirement up to four liters by nasal cannula. Laboratory evaluation demonstrated leukocytosis with a white blood cell (WBC) count of 14.4 × 10^9^/L (reference range: 3.4–9.6 × 10^9^/L. All reference ranges below reflect institutional standards), hyperbilirubinemia (6.6 mg/dL—reference range: 0.0–1.2 mg/dL), and significant lactic acidosis (lactate 5.1 mmol/L—reference range: 0.5–2.2 mmol/L), consistent with severe sepsis. Blood cultures were obtained, and although *Salmonella* species were not specifically suspected, empiric antimicrobial therapy with intravenous ceftriaxone and vancomycin was initiated along with aggressive fluid resuscitation.

A comprehensive evaluation was undertaken to identify a primary infectious source and assess for potential metastatic complications. Nasopharyngeal respiratory pathogen testing using polymerase chain reaction (PCR) was negative. Chest radiography demonstrated vascular and interstitial prominence without focal consolidation. Urinalysis was without significant pyuria. Abdominal ultrasound revealed gallbladder sludge without evidence of cholecystitis. Computer tomography (CT) angiography of the chest showed parenchymal opacities interpreted as chronic scarring from prior acute chest syndrome or pneumonia rather than acute infection, and CT of the abdomen and pelvis with intravenous contrast demonstrated no intra-abdominal source. Plain radiographs of the right shoulder and knee showed known avascular necrosis of the humeral head and possible bone infarcts without evidence of acute osseous destruction. Initial blood cultures subsequently grew Gram-negative bacilli in both aerobic and anaerobic sets at 8.45 and 8.55 h, consistent with high-grade bacteremia.

On the morning of hospital day 3, the patient experienced further clinical worsening with increasing somnolence, escalating oxygen requirements, and persistent tachycardia. Repeat laboratory testing revealed uptrending leukocytosis (WBC 22.4 × 10^9^/L), lactic acidosis (lactate 5.7 mmol/L), and rapidly rising hyperbilirubinemia (total bilirubin 13.1 mg/dL), reflecting progressive sepsis. Repeat blood cultures and the stool gastrointestinal pathogen PCR panel were obtained. Antimicrobial therapy was broadened to piperacillin–tazobactam 3.375 g IV every 6 h in conjunction with vancomycin 1.75 g every 8 h; additional intravenous fluids were administered. Over the ensuing hours, lactic acidosis improved; however, the patient remained intermittently febrile to a Tmax of 39.9 °C, tachycardic, and oxygen-dependent. His course was further complicated by severe anemia related to suspected hemolysis and vaso-occlusion, with limited transfusion options due to his status as a Jehovah’s Witness.

**Figure 1:**
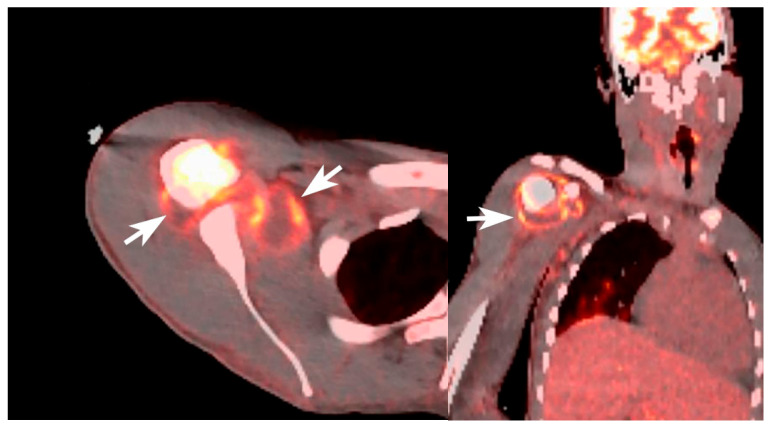
Axial and coronal PET-CT images demonstrating abnormal hypermetabolism involving the proximal right humerus and peripheral hypermetabolism surrounding the joint effusion. Arrows point to the inflamed (hypermetabolic) synovium surrounding the joint effusion in the setting of septic arthritis.

**Figure 2:**
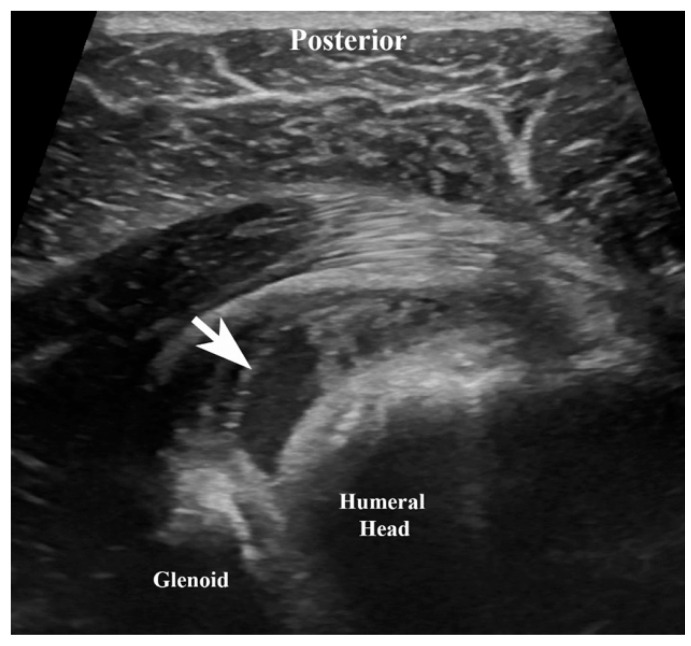
A single ultrasound image depicts the joint effusion targeted for aspiration. This is a view from the posterior shoulder with the arrow pointing to the joint fluid/effusion. The humeral head and glenoid are labeled for anatomic reference.

Despite broad-spectrum antimicrobial therapy, the patient had persistent fever and bacteremia without an identifiable source on initial imaging, raising concern for metastatic infection. Repeat blood cultures remained positive in one of two sets after 67 h. The gastrointestinal pathogen PCR panel returned positive for *Salmonella* species, supporting enteric acquisition, and blood cultures from admission ultimately speciated to pan-susceptible *Salmonella enterica* on hospital day 5, eventually clearing on hospital day 7. Given the high risk of hematogenous dissemination in the setting of SCD and sustained bacteremia, advanced imaging was pursued to identify occult infectious foci. Whole-body Positron Emission Tomography (PET)-CT imaging demonstrated moderate hypermetabolism involving the proximal right humerus and the periphery of a lobulated fluid collection within the right shoulder (Figure 1). Ultrasound-guided arthrocentesis of the right shoulder (Figure 2) was performed on hospital day 9, yielding inflammatory synovial fluid with a 74,783 leukocyte count reflecting a 95% neutrophilic predominance. Cultures grew *Salmonella* species with an identical susceptibility profile, confirming hematogenous seeding of the native joint and resulting septic arthritis. Aspiration and magnetic resonance imaging (MRI) of the right knee were not consistent with infection, and cultures remained negative.

The patient subsequently underwent arthroscopic irrigation and debridement of the right shoulder on hospital day 10 without complications. Following surgical source control and availability of antimicrobial susceptibility data (Table 1), antimicrobial therapy was narrowed to intravenous ceftriaxone 2 g once daily with a planned six-week course. The patient demonstrated sustained clinical improvement with resolution of fever and was discharged to a subacute rehabilitation facility on hospital day 18 to complete antimicrobial therapy and physical rehabilitation. A detailed timeline summarizing key clinical events, diagnostic studies, microbiologic findings, and therapeutic interventions during the hospitalization is provided in Table 2.

## 3. Discussion

Non-typhoidal *Salmonella* (NTS) species are well-recognized causes of severe infection in patients with sickle cell disease (SCD), with a particular predilection for the musculoskeletal system [1,4,8]. Although *Salmonella* osteomyelitis is well described in this population, *Salmonella* septic arthritis is considerably less common and has been reported primarily in isolated case reports and small case series [2,4,15]. Hematogenous seeding of a native shoulder joint has been described only rarely, particularly among young adults with SCD.

We report a rare manifestation of NTS (*Salmonella enterica*) bacteremia characterized by persistent bloodstream infection with hematogenous dissemination to a native shoulder joint, complicated by acute chest syndrome and marked functional decline. This case highlights the unique pathogenic interplay between *Salmonella* species and sickle cell disease, resulting in an uncommon and severe pattern of invasive infection.

### 3.1. Review of the Literature

We conducted a literature review to identify published reports of NTS-caused septic arthritis. Database searches were performed using the terms “*Salmonella*,” “non-typhoidal *Salmonella*,” and “*Salmonella enterica*,” in combination with “septic arthritis,” “joint infection,” “septic joint,” and “sickle cell disease.” Studies were included if they reported microbiologic identification of NTS from synovial fluid, blood, or tissue specimens (Table 3).

Septic arthritis due to NTS is a rare clinical entity. Published reports demonstrate a predilection for weight-bearing joints, particularly the hip and knee, whereas shoulder involvement remains uncommon. Although SCD is a well-established risk factor for *Salmonella* osteomyelitis, only a minority of reported cases of *Salmonella* septic arthritis involve patients with SCD [7,16]. Diagnostic confirmation in these reports typically relies on synovial fluid cultures, often supported by advanced imaging such as magnetic resonance imaging, with blood cultures positive in cases complicated by concomitant bacteremia.

Management strategies across reported cases are relatively consistent and generally include prolonged intravenous antimicrobial therapy, most commonly third-generation cephalosporins or fluoroquinolones, combined with surgical intervention ranging from joint aspiration to arthroscopic or open incision and debridement. In cases involving prosthetic joints, revision surgery is often required. Reported cases of shoulder involvement are particularly limited and frequently occur outside the context of SCD or in the presence of additional predisposing factors, such as immunosuppression in patients with systemic lupus erythematosus [8]. In contrast, more commonly reported causes of septic arthritis in patients with SCD include *Staphylococcus aureus* and *Staphylococcus epidermidis* [15].

Ghazanfar et al. described a case of *Salmonella* septic arthritis involving the native shoulder and elbow in a patient with SCD; however, *Salmonella* was isolated only from blood cultures, and synovial fluid cultures demonstrated no growth [16]. In contrast, our case describes a young adult with SCD who developed persistent *Salmonella enterica* bacteremia with hematogenous seeding of a native shoulder joint. This case underscores the rarity of *Salmonella* shoulder septic arthritis in SCD and expands the literature by highlighting an atypical anatomic site, a well-defined pathogenesis, and successful management with arthroscopic debridement and prolonged targeted antimicrobial therapy.

### 3.2. Epidemiology of Salmonella Infections in SCD

Patients with SCD are predisposed to invasive *Salmonella* infections due to functional asplenia, impaired opsonization, complement dysfunction, and recurrent ischemia–reperfusion injury leading to microvascular infarction of bone [1]. Multiple studies have demonstrated that *Salmonella* species account for a substantial proportion of osteomyelitis cases in patients with SCD, in some series exceeding those of *Staphylococcus aureus*, particularly in pediatric populations [2,4,5,6].

In contrast, *Salmonella* septic arthritis is reported far less frequently, and its true incidence is likely underestimated because of diagnostic challenges related to clinical overlap with vaso-occlusive pain crises [7]. Among the reported cases in patients with SCD, involvement most commonly affects large, weight-bearing joints such as the hip and knee [7,15]. Shoulder involvement remains uncommon and, when described, is more often associated with prosthetic joints, traumatic inoculation, or additional immunosuppressive conditions in patients with SCD [8,9,21].

Native shoulder septic arthritis caused by *Salmonella* in an adult with SCD, as observed in our patient, therefore represents a rare and atypical clinical presentation.

### 3.3. Pathophysiology of Salmonella Septic Arthritis

This patient’s susceptibility to *Salmonella* septic arthritis can be explained by a multistep hematogenous seeding process that is characteristic of the SCD phenotype.

#### 3.3.1. Gastrointestinal Breach and Bacterial Translocation

The gastrointestinal tract serves as the primary reservoir for *Salmonella* species, with foodborne exposure, most commonly through the consumption of undercooked poultry, representing a well-established route of infection [13]. In this case, the patient’s recent ingestion of undercooked chicken was identified as the most likely source of NTS infection, consistent with well-described epidemiologic associations between poultry products and *Salmonella enterica* [13].

In patients with SCD, recurrent vaso-occlusive episodes lead to microinfarction of the intestinal mucosa, resulting in ischemic injury, increased intestinal permeability, disruption of the microbiome, and chronic inflammation. These pathophysiologic changes compromise the gut–blood barrier and facilitate bacterial translocation into the systemic circulation. Because *Salmonella* can survive intracellularly within macrophages, patients with SCD, who have impaired reticuloendothelial function, are particularly susceptible to persistent bacteremia once translocation occurs [10,12].

Although direct biomarkers of intestinal permeability or microbiome disruption were not assessed in this case, the proposed mechanism of bacterial translocation is supported by the established literature describing gut barrier dysfunction in sickle cell disease [10,11,12].

#### 3.3.2. Reticuloendothelial Dysfunction and Functional Asplenia

Under normal conditions, the spleen plays a critical role in clearing encapsulated and intracellular pathogens, including *Salmonella*. However, most patients with SCD develop functional asplenia or autosplenectomy as a consequence of repeated splenic infarction. The resulting loss of phagocytic filtration and impaired opsonization allows sustained bacteremia and increases the likelihood of hematogenous seeding of distant sites, including native joints [3,22].

#### 3.3.3. Iron Dysregulation and Gram-Negative Virulence in Sickle Cell Disease

An additional contributor to the severity and persistence of invasive *Salmonella* infection in SCD is dysregulated iron homeostasis. Gram-negative pathogens, including *Salmonella enterica*, depend on iron for growth and virulence, and host “nutritional immunity” limits microbial replication by restricting iron availability [23]. In SCD, chronic intravascular hemolysis and inflammatory stress can increase circulating heme and non-transferrin-bound iron, potentially creating a permissive milieu that favors iron-dependent bacterial proliferation [23]. In parallel, *Salmonella* has evolved iron-scavenging strategies that allow it to compete successfully even in inflamed host environments where iron is actively sequestered [24]. Mechanistically, infection-associated heme oxygenase-1 signaling can modulate macrophage antimicrobial effector pathways during *Salmonella* infection, influencing early bacterial control and potentially contributing to persistence in susceptible hosts [25]. In this patient, severe hemolysis with marked hyperbilirubinemia during acute illness plausibly increased bioavailable heme/iron and, together with impaired reticuloendothelial clearance, may have contributed to sustained bacteremia and an increased risk of hematogenous dissemination to the shoulder joint.

#### 3.3.4. The Infarct-Infection Niche Within Bone

A leading explanation for *Salmonella*’s predilection for the musculoskeletal system in SCD involves the bone marrow microenvironment. Vaso-occlusive crises result in areas of bone infarction characterized by sluggish blood flow, hypoxia, and local tissue necrosis. These regions represent a locus minoris resistentiae, providing a protected niche in which bacteria can lodge, evade host immune defenses, and proliferate. Extension of the infarcted bone into the adjacent joint space may subsequently lead to septic arthritis. In this case, the patient’s history of bilateral humeral head avascular necrosis likely created a permissive environment for hematogenous seeding of the native shoulder joint [14,26]. When considered alongside systemic factors, including persistent bacteremia, functional asplenia, and impaired reticuloendothelial clearance, this combination provides a unifying explanation for preferential hematogenous localization to the glenohumeral joint.

### 3.4. Diagnostic Challenges in Sickle Cell Disease and the Role of Advanced Imaging

Distinguishing septic arthritis from vaso-occlusive crisis represents a major diagnostic challenge in patients with SCD. Both conditions frequently present with overlapping clinical features, including severe localized pain, joint swelling, restricted range of motion, leukocytosis, and elevated inflammatory markers, and there is no definitive diagnostic gold standard to reliably differentiate between them [27,28,29]. Fever may be absent early in the disease course, and baseline anemia and ongoing hemolysis can further complicate the interpretation of laboratory findings. Consequently, septic arthritis may be underrecognized or diagnosed late in patients with SCD, potentially resulting in delayed treatment and worse clinical outcomes [29,30].

In this case, the patient was initially admitted for a vaso-occlusive pain crisis with severe knee and shoulder pain. Subsequent clinical deterioration accompanied by persistent bacteremia raised concern for an infectious etiology and prompted further diagnostic evaluation. Advanced imaging modalities, including positron emission tomography–computed tomography (PET-CT) and magnetic resonance imaging (MRI), in conjunction with diagnostic arthrocentesis, were instrumental in identifying septic arthritis of the shoulder while excluding infection in other painful joints.

MRI is a particularly valuable imaging modality in SCD, as it can detect septic arthritis and often demonstrates infection extending beyond the joint space into adjacent bone or soft tissue. PET-CT is a useful tool for identifying occult infectious foci in patients with culture-proven bacteremia, with reported sensitivities of 90.9% and specificities of 87.5%, especially when conventional imaging studies are inconclusive or negative [31,32,33].

In retrospect, several features favored an infectious etiology over a routine vaso-occlusive crisis. These included progressive systemic deterioration with high-grade fevers, persistent bacteremia, and hemodynamic instability, which are atypical for uncomplicated vaso-occlusive episodes. Additionally, sustained leukocytosis, rising lactate, and evolving localized joint findings raised suspicion for septic arthritis. The persistence and progression of symptoms despite supportive care further supported an alternative diagnosis.

### 3.5. Management Strategies

Management of *Salmonella* septic arthritis in patients with SCD requires a combined approach of prompt source control and prolonged antimicrobial therapy. Given the high risk of invasive infection and diagnostic uncertainty in this population, early initiation of empiric broad-spectrum antimicrobial therapy is warranted, followed by prolonged pathogen-directed treatment once microbiologic data are available. Treatment durations of four to six weeks are typically recommended for septic arthritis in this setting.

Early joint aspiration or tissue sampling is critical when clinical suspicion for infection is high, as laboratory and clinical findings alone are often insufficient to reliably distinguish septic arthritis from vaso-occlusive crisis [29]. When septic arthritis is confirmed or strongly suspected, timely surgical intervention, via arthroscopic or open irrigation and debridement, has been associated with improved outcomes, particularly when performed early in the disease course [34,35]. In this case, the patient underwent arthroscopic irrigation and debridement of the right shoulder, with synovial fluid cultures confirming *Salmonella* species, consistent with current management principles.

Antimicrobial regimens reported in the literature vary with susceptibility patterns; however, third-generation cephalosporins and fluoroquinolones are the most frequently used agents [17,36]. In this patient, antimicrobial therapy was appropriately escalated and subsequently de-escalated in response to culture data and clinical improvement, with eventual transition to ceftriaxone for a planned six-week course. Bacteremia clearance was confirmed by repeat negative blood cultures.

### 3.6. Ethical and Other Management Considerations

This patient’s status as a Jehovah’s Witness introduced unique challenges in the management of severe sickle cell crisis and sepsis-associated hemolysis, as blood transfusion options were limited. In such cases, multidisciplinary planning involving hematology, infectious diseases, orthopedic surgery, anesthesiology, and ethics consultation is essential to balance effective infection management and surgical intervention while respecting patient autonomy and religious beliefs.

An often-underrecognized aspect of septic arthritis in patients with SCD is the substantial functional morbidity associated with invasive infection, surgical management, prolonged hospitalization, pain, and physical deconditioning. In this case, septic arthritis resulted in significant impairments in mobility and activities of daily living, ultimately necessitating inpatient rehabilitation. Although functional outcomes are infrequently reported in prior case reports, they represent an important consideration, particularly in young adults with SCD who often have preexisting musculoskeletal limitations related to avascular necrosis and cumulative disease burden [34].

## 4. Conclusions

This case illustrates a rare presentation of non-typhoidal *Salmonella enterica* septic arthritis involving a native shoulder joint in a young adult with SCD, complicated by persistent bacteremia, acute chest syndrome, and significant functional morbidity. The clinical course underscores the importance of thorough and detailed history taking in patients with SCD who present with focal musculoskeletal pain, as exposure history, such as recent ingestion of undercooked poultry, may provide critical clues to the underlying pathogen and inform early diagnostic and therapeutic decision-making. Additionally, persistent bacteremia despite appropriate antimicrobial therapy should prompt a comprehensive evaluation for metastatic foci of infection, including the targeted use of advanced imaging modalities.

Recognition of these principles may facilitate earlier diagnosis, timely source control, and improved functional recovery in patients with sickle cell disease who develop invasive *Salmonella* infections.

## Figures and Tables

**Table 1:** Antimicrobial susceptibility of *Salmonella enterica* isolated from deep tissue samples.

Antimicrobial Agent	MIC (μg/mL)	Interpretation
Ampicillin	≤2	Susceptible
Ceftriaxone	≤0.25	Susceptible
Ciprofloxacin	≤0.06	Susceptible
Levofloxacin	≤0.12	Susceptible
Trimethoprim + Sulfamethoxazole	≤20	Susceptible

**Table 2:** Clinical timeline of hospital course, diagnostic evaluation, and management.

Date (2025)	Hospital Day	Clinical Events
Pre-admission		Consumption of undercooked poultry in a patient with homozygous sickle cell disease and functional asplenia.
23 November	0	Admission for vaso-occlusive pain crisis involving the right upper extremity and bilateral knees; afebrile without focal infectious symptoms.
25 November	2	Acute deterioration with fever, rigors, hypotension, tachycardia, and new oxygen requirement. Laboratory evaluation notable for leukocytosis (14.1 × 10^9^/L) and lactic acidosis (5.1 mmol/L). Blood cultures obtained; empiric ceftriaxone and vancomycin initiated.
25–26 November	2–3	Broad infectious evaluation performed without identification of a primary source; imaging demonstrated chronic changes and known avascular necrosis only.
26 November	3	Worsening encephalopathy and respiratory status. Laboratory studies revealed leukocytosis (22.4 × 10^9^/L), rising lactate (5.7 mmol/L), hyperbilirubinemia, transaminitis, and elevated NT-proBNP. Antimicrobial therapy broadened to piperacillin–tazobactam.
28 November	5	Blood cultures speciated to pan-susceptible *Salmonella enterica*. Gastrointestinal pathogen PCR positive for *Salmonella* species.
29 November	6	Repeat blood cultures positive in 1 of 2 sets after 67 h, raising concern for metastatic infection.
30 November	7	Blood cultures cleared.
1 December	7–8	Whole-body PET-CT demonstrated hypermetabolic activity in the proximal right humerus with adjacent shoulder fluid collection.
2 December	9	Ultrasound-guided arthrocentesis of the right shoulder performed; synovial fluid cultures grew *Salmonella* species.
3 December	10	Arthroscopic irrigation and debridement of the right shoulder performed without complication. Blood cultures remained negative.
3 December onward	—	Antimicrobial therapy narrowed to intravenous ceftriaxone with a planned six-week course.
11 December	—	Discharged to a subacute rehabilitation facility with resolution of bacteremia and sustained clinical improvement.

**Table 3:** Studies reporting septic arthritis caused by non-typhoidal Salmonella.

Author, Year	Patient	Joint (Type)	Sickle Cell Disease	Co-Morbidity/Concomitant Musculoskeletal Involvement	Diagnosis	Management
Henderson RC et al., 1989 [7]	26 yr/M	Knee (Native)	Yes	-	Joint aspirate culture positive for *Salmonella*	-
Ghazanfar H et al., 2021 [16]	28 yr/F	Shoulder and Elbow (Native)	Yes	Osteomyelitis & pyomyositis	MRI+ Joint aspirate cultures No Growth. Blood culture positive for *Salmonella* sp.	IV Ceftriaxone + Surgical arthroscopic incision and drainage
Yu D et al., 2024 [8]	43 yr/F	Shoulder (Native)	No	SLE	Aspirate joint fluid culture positive for NT-Salmonella	IV ceftriaxone + surgical debridement due to worsening
Al Nafeesah AS, 2015 [17]	11 m/F	Elbow (Native)	No	No	USG + Aspirate joint fluid culture positive for NT-Salmonella group D	IV Cefotaxime + elbow arthrotomy with washout
Howes M et al., 2020 [18]	5 yr/F	Hip (Native)	No	No	MRI + Joint aspiration culture positive for *S. agona*	Fluoroquinolone therapy
Jiang B et al., 2023 (Case series) [19]	53 yr median (range 15–56)	Hip (Native)	No	Variable	Aspirate joint fluid culture positive for *S. enterica Dublin serotype*	IV Antibiotics + surgical management
Gharib MH et al., 2022 [20]	37 yr/M	Hip (Native)	No	No	MRI + synovial fluid culture growing *Salmonella B*	IV Ceftriaxone + surgical arthrotomy with washout
Santoso A et al., 2020 [9]	67 yr/F	Hip (Prosthetic)	No	No	Wound fistula and joint tissue culture positive for NT-Salmonella	IV Ciprofloxacin + Two-stage revision
Present case	22 yr/M	Shoulder (Native)	Yes	AVN of rt. femoral head and b/l humeral head	Blood culture + Joint aspiration culture positive for *Salmonella enterica*	IV Ceftriaxone (6 wk) + Arthroscopic irrigation and debridement

## Data Availability

No new data were created or generated in this case report. All information presented is derived from the patient’s clinical course and existing medical records, and no publicly archived datasets were analyzed. Data sharing does not apply to this article.
